# Supplementary material for: Biocompatible Polymer-Grafted TiO2 Nanoparticle Sonosensitizers Prepared Using Phosphonic Acid-Functionalized RAFT Agent
Source: Polymers (Basel). 2023 May 23;15(11):2426. doi: 10.3390/polym15112426 (PMC10255893; doi:10.3390/polym15112426)
Supplement: Supplementary file 1 [file polymers-15-02426-s001.zip › polymers-2337996-supplementary.pdf]

Supplementary Materials: Biocompatible Polymer-Grafted TiO_2 Nanoparticle Sonosensitizers Prepared Using Phosphonic Acid-Functionalized RAFT Agent

Yukiya Kitayama [1,2], Aoi Katayama [2], Zhicheng Shao [1] and Atsushi Harada [1,2,*]

1 Department of Applied Chemistry, Graduate School of Engineering, Osaka Prefecture University, 1-1 Gakuen-cho, Naka-ku, Sakai, Osaka 599-8531, Japan; kitayama@omu.ac.jp (Y.K.); shao.zhicheng.36a@st.kyoto-u.ac.jp (Z.S.)
2 Department of Applied Chemistry, Graduate School of Engineering, Osaka Metropolitan University, 1-1 Gakuen-cho, Naka-ku, Sakai, Osaka 599-8531, Japan; sc22139u@st.omu.ac.jp
* Correspondence: atsushi_harada@omu.ac.jp

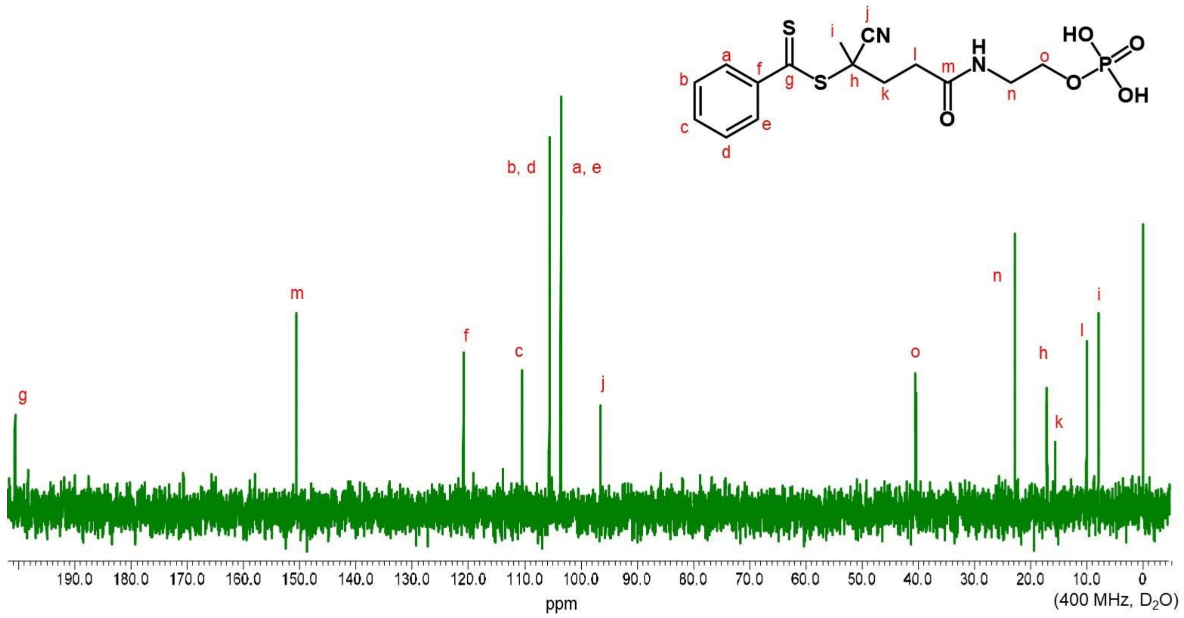

Figure S1. ^{13}C NMR spectrum of RAFT-PO_4H_2 in D_2O.

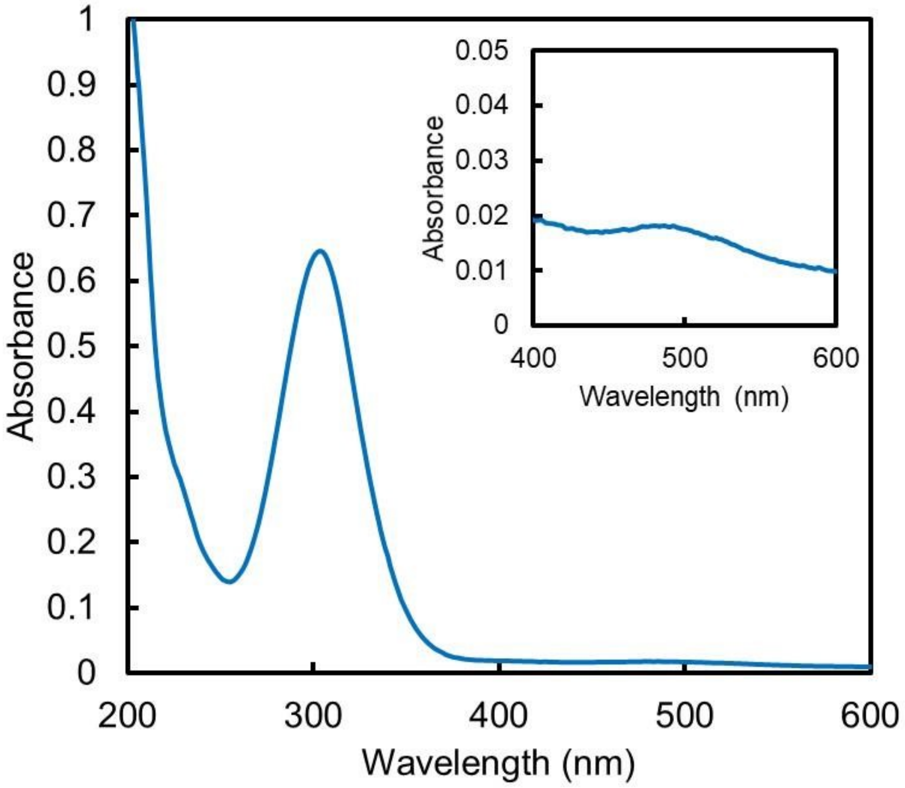

Figure S2. UV-Vis spectrum of RAFT-PO_4H_2.

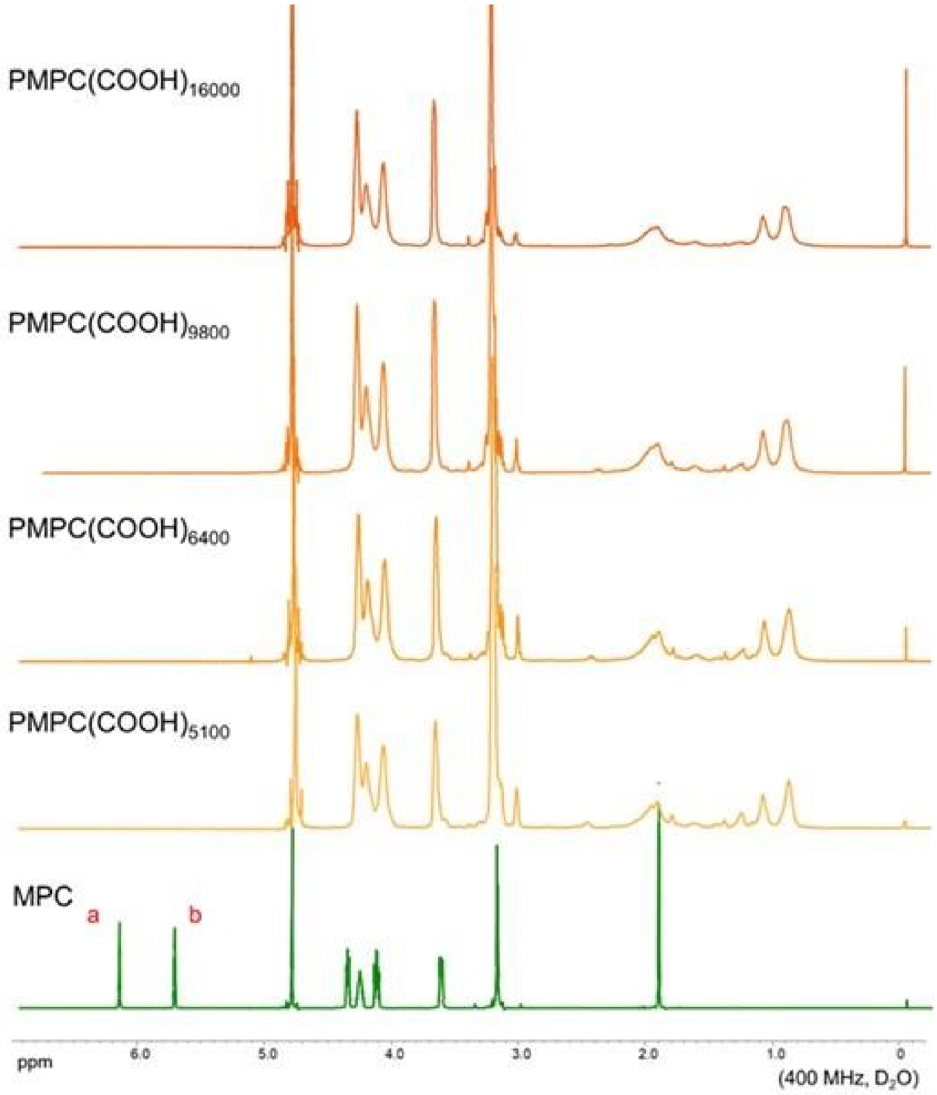

Figure S3. ^{1}H NMR spectra of MPC and polymer solutions obtained after RAFT polymerizations of MPC using RAFT-COOH with different molar ratio of MPC and RAFT-PO_4H_2.

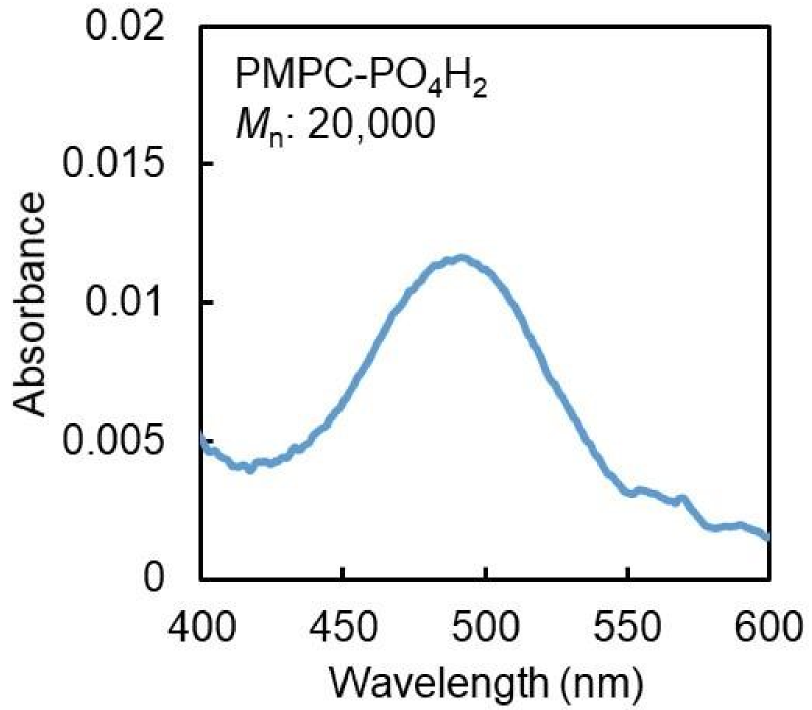

Figure S4. UV-Vis spectra of PMPC-PO_4H_2 prepared with RAFT- PO_4H_2. Polymer concentration: 10 mg/mL. Solvent: Water dissolving Brij98 (0.5 mM)

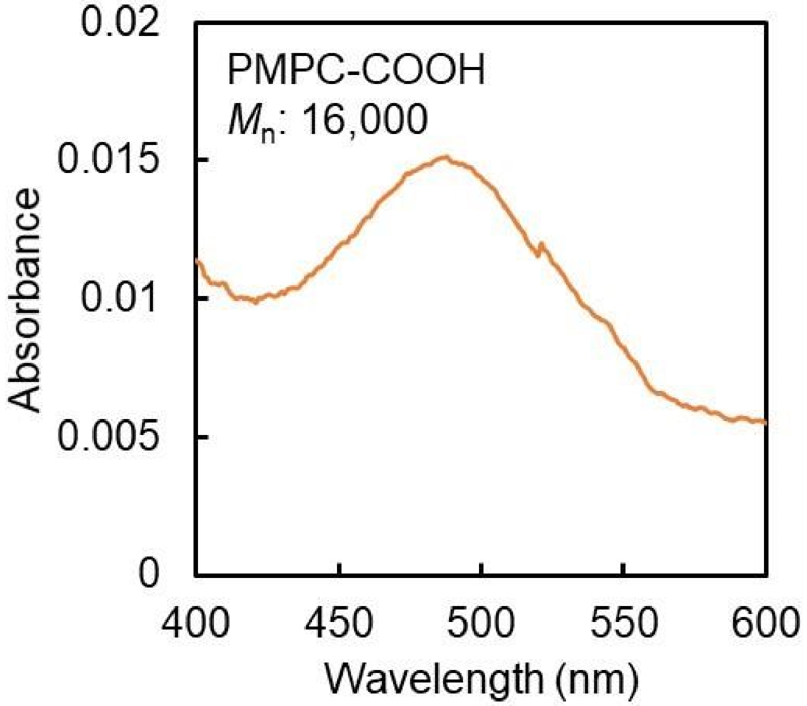

Figure S5. UV-Vis spectra of PMPC-COOH prepared with RAFT-COOH. Polymer concentration: 10 mg/mL. Solvent: Water dissolving Brij98 (0.5 mM).

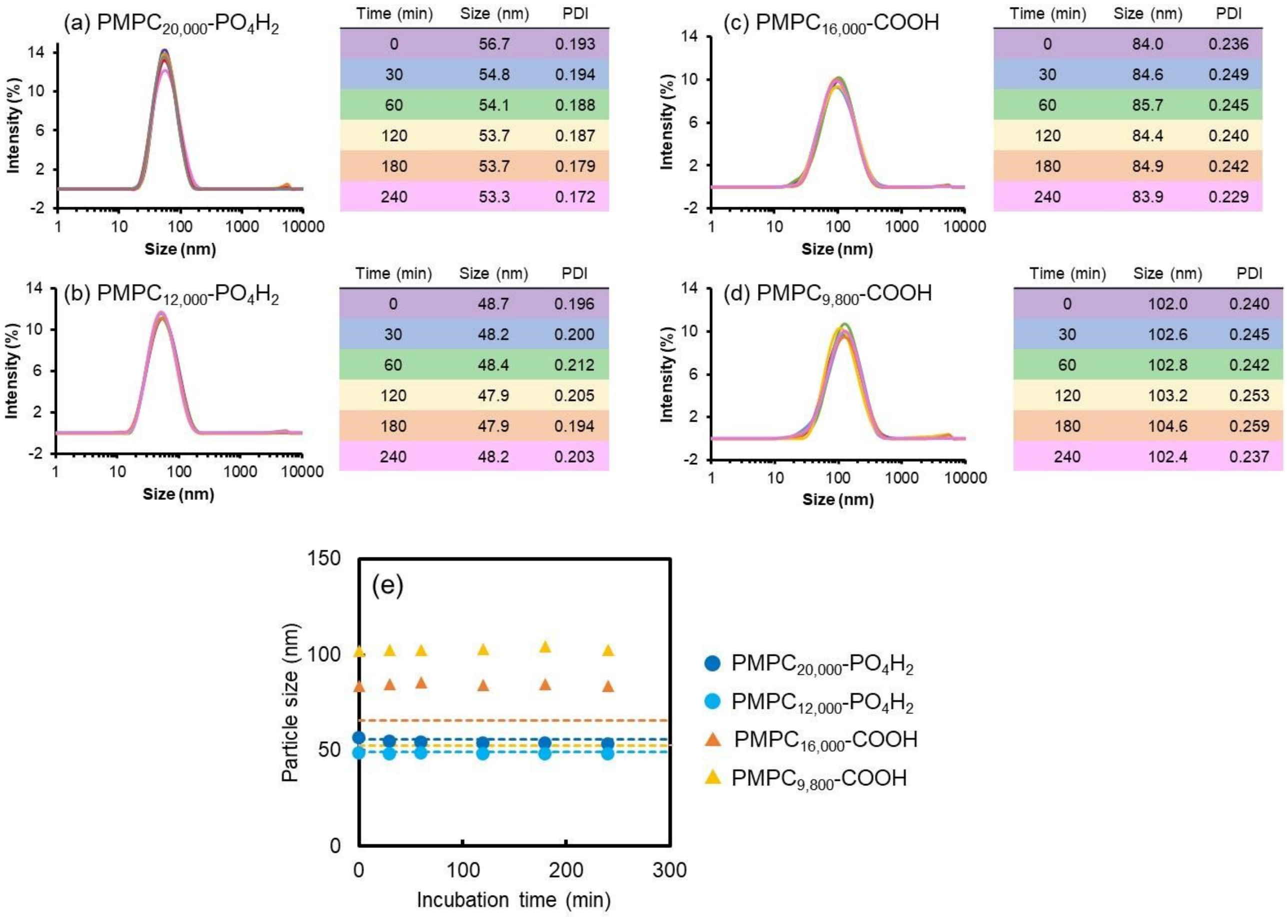

(a)

Time (min)	Size (nm)	PDI
0	56.7	0.193
30	54.8	0.194
60	54.1	0.188
120	53.7	0.187
180	53.7	0.179
240	53.3	0.172

(b)

Time (min)	Size (nm)	PDI
0	48.7	0.196
30	48.2	0.200
60	48.4	0.212
120	47.9	0.205
180	47.9	0.194
240	48.2	0.203

(c)

Time (min)	Size (nm)	PDI
0	84.0	0.236
30	84.6	0.249
60	85.7	0.245
120	84.4	0.240
180	84.9	0.242
240	83.9	0.229

(d)

Time (min)	Size (nm)	PDI
0	102.0	0.240
30	102.6	0.245
60	102.8	0.242
120	103.2	0.253
180	104.6	0.259
240	102.4	0.237

Figure S6. Particle size distributions, average particle size and PDI values of PMPC-modified TiO_2 nanoparticles prepared with PMPC$_{20000}$-PO$_4$H$_2$ (a), PMPC$_{12000}$-PO$_4$H$_2$ (b), PMPC$_{12,000}$-COOH (c), and PMPC$_{9,800}$-COOH (d) at different incubation times (0 min: purple, 30 min: blue, 60 min: green, 120 min: yellow, 180 min: orange, 240 min: pink) in pure water. Particle size v.s. incubation time plots of PMPCmodified TiO_2 nanoparticles prepared with PMPC$_{20000}$-PO$_4$H$_2$, PMPC$_{12000}$-PO$_4$H$_2$, PMPC$_{12,000}$-COOH, and PMPC$_{9,800}$-COOH after 100 times dilution with pure water (e). Dashed lines in (e) are particle size before dilution.

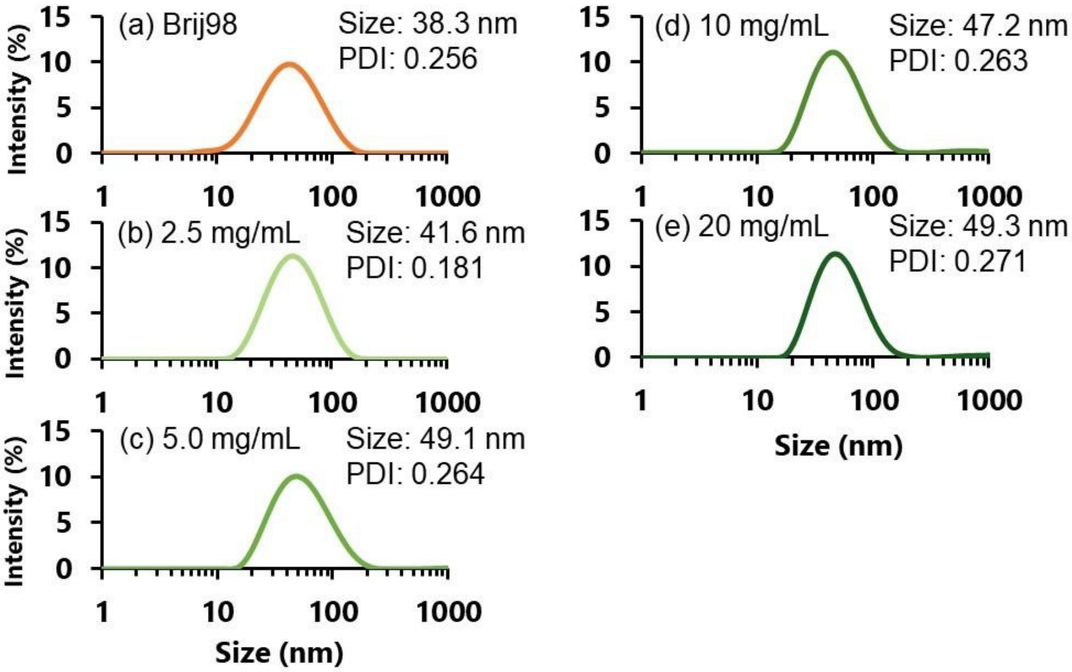

Figure S7. Particle size distributions of Brij98-stabilized (a) and PMPC-modified TiO_2 nanoparticles prepared with different concentrations of $PMPC_{7600}$-PO_4H_2 (b: 2.5, c: 5.0, d: 10, e: 20 mg/mL).
